# Activation-induced cytidine deaminase is a possible regulator of cross-talk between oocytes and granulosa cells through GDF-9 and SCF feedback system

**DOI:** 10.1038/s41598-021-83529-x

**Published:** 2021-02-15

**Authors:** Takashi Iizuka, Kousho Wakae, Masanori Ono, Takuma Suzuki, Yasunari Mizumoto, Kouichi Kitamura, Shin-ichi Horike, Masamichi Muramatsu, Hiroshi Fujiwara

**Affiliations:** 1 Department of Obstetrics and Gynecology, Graduate School of Medical Sciences, Kanazawa University, Takara-machi 13-1, Kanazawa, Ishikawa 920-8640 Japan; 2 Department of Virology II, National Institute of Infectious Diseases, Tokyo, 162-8640 Japan; 3 Advanced Science Research Center, Kanazawa University, Kanazawa, Japan

**Keywords:** Developmental biology, Physiology

## Abstract

Activation-induced cytidine deaminase (AID, Aicda) is a master gene regulating class switching of immunoglobulin genes. In this study, we investigated the significance of AID expression in the ovary. Immunohistological study and RT-PCR showed that AID was expressed in murine granulosa cells and oocytes. However, using the Aicda-Cre/Rosa-tdRFP reporter mouse, its transcriptional history in oocytes was not detected, suggesting that AID mRNA in oocytes has an exogenous origin. Microarray and qPCR validation revealed that mRNA expressions of growth differentiation factor-9 (GDF-9) in oocytes and stem cell factor (SCF) in granulosa cells were significantly decreased in AID-knockout mice compared with wild-type mice. A 6-h incubation of primary granuloma cells markedly reduced AID expression, whereas it was maintained by recombinant GDF-9. In contrast, SCF expression was induced by more than threefold, whereas GDF-9 completely inhibited its increase. In the presence of GDF-9, knockdown of AID by siRNA further decreased SCF expression. However, in AID-suppressed granulosa cells and ovarian tissues of AID-knockout mice, there were no differences in the methylation of SCF and GDF-9. These findings suggest that AID is a novel candidate that regulates cross-talk between oocytes and granulosa cells through a GDF-9 and SCF feedback system, probably in a methylation-independent manner.

## Introduction

The apolipoprotein B mRNA editing enzyme, catalytic polypeptide-like (APOBEC) family is a group of cytidine deaminases that convert cytosine (C) to uracil (U) in DNA/RNA^1^. In humans, the APOBEC family is composed of 11 members (AID and APOBEC1, 2, 3A, 3B, 3C, 3D, 3F, 3G, 3H and 4). In mice, 4 types have been confirmed (AID and APOBEC 1–3). AID is abundantly expressed in mammalian B lymphocytes, and induces genetic modification in antibody genes. In B lymphocytes, AID is involved in class switching that induces DNA cleavage by generating uracil in antibody genes. AID can also act on the variable region of the antibody gene, inducing hypermutation and altering the affinity of the antibody for its antigen^2^.

Ectopic expression of AID in non-lymphoid tissues is induced by inflammation. AID leads to the accumulation of mutations and is associated with carcinogenesis in fallopian tubes^3^, gastric mucosa^4^, and skin^5^. On the other hand, AID is expressed in normal mouse ovaries^6–8^, the genital ridge^6^, and primordial germ cells^9^. It has also been demonstrated that the estrogen-estrogen receptor complex binds to the Aicda promoter, producing AID protein both in lymphoid and non-lymphoid organs including ovaries^10^. Nonetheless, as AID-knockout (KO) mice showed no fertility abnormal phenotype^7,8^, its significance, as well as distribution, remains to be determined.

Our preliminary examination confirmed that immunoreactive AID is highly expressed in cumulus granulosa cells^7^. Since oocytes should be protected from genetic mutation, it is curious that the mutagenic enzyme AID is present around oocytes. Recently, it was reported that follicular fluid contained extracellular vesicles (EVs) and follicular EVs include granulosa cell-derived mRNA^11^, suggesting a new mechanism for communication within the ovarian follicle and the cross-talk between oocytes and cumulus granulosa cells through EVs^12–15^. In addition, exogenously labelled-AID microinjected into the cytoplasm of oocytes in the germinal vesicle stage was shown to enter the nucleus of oocytes just before germinal vesicle breakdown, suggesting that AID can directly access oocyte DNA^16^. Since AID may play a role in epigenetic reprogramming^6,17,18^, it is necessary to clarify the physiological roles of AID in the cross-talk between oocytes and cumulus granulosa cells. Thus, we examined the expression profiles of AID in the mouse ovary and its possible role in ovarian functions.

## Results

### AID is expressed in murine oocytes and granulosa cells

**Figure 1:**
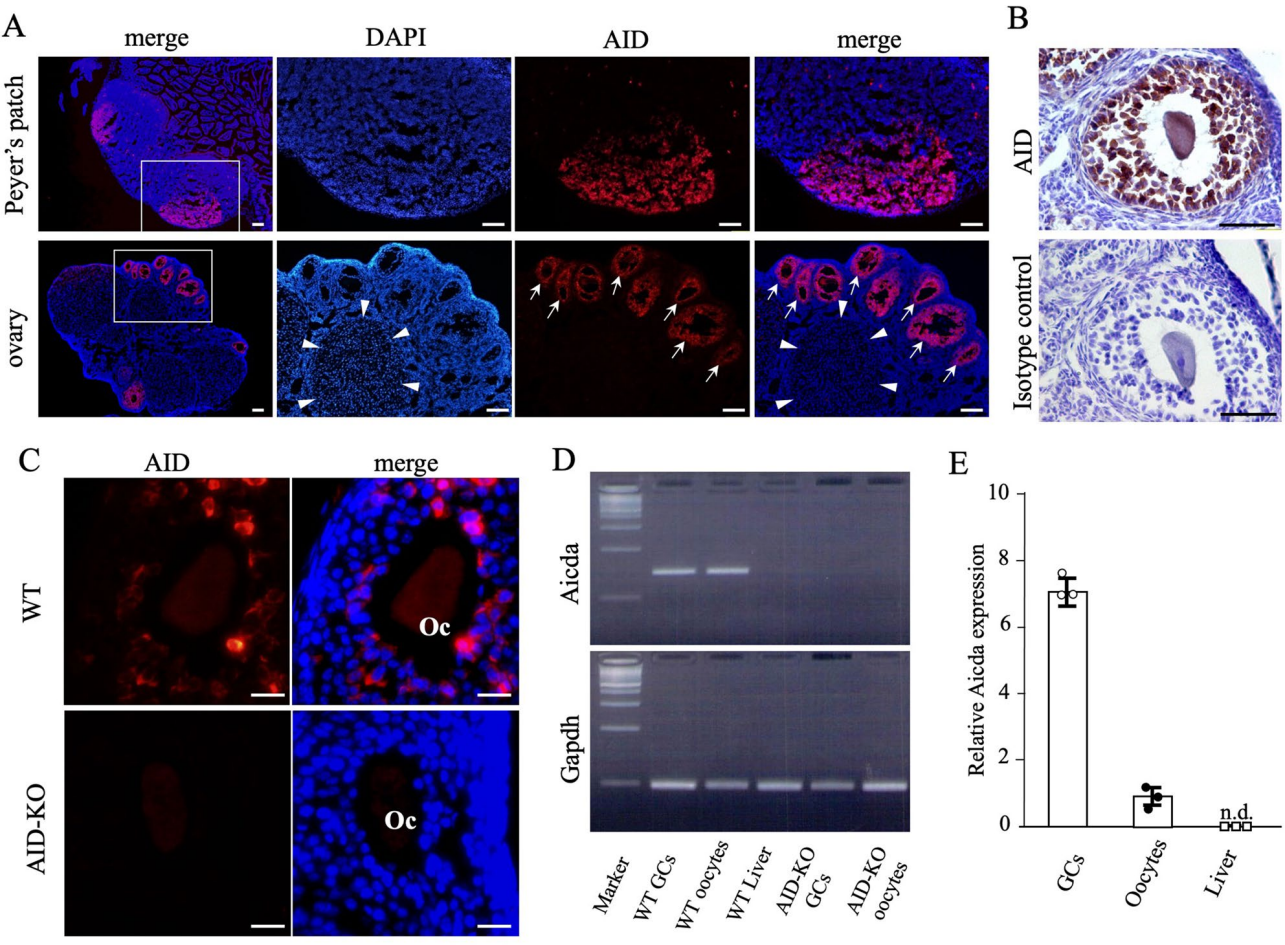
The expression profiles of AID protein in Peyer’s patches and ovaries. (**A**) Immunofluorescence of AID in 8-week-old mouse Peyer’s patches and ovaries. The right 3 panels show magnified views of the white square areas outlined in the left panel. AID was expressed in the germinal center of Peyer’s patches. Of note, AID was expressed only in follicles (arrows) and not expressed in the corpus luteum (arrowheads). Bars show 100 μm. (**B**) Immunohistochemical staining of AID in mouse follicles. Immunoreactive AID was observed in granulosa cells and oocytes. (**C**) AID staining of the ovaries using a different antibody in wild-type and AID-KO mice. AID was also observed in granulosa cells and oocytes (Oc), whereas no expression of AID was observed in the follicles of AID-KO mice. Bars show 20 μm. (**D**) RT-PCR analysis of AID and Gapdh mRNA expression. The mRNA expression of AID in granulosa cells and oocytes was confirmed. The data shown are representative of two independent experiments. Full-length gels are presented in Supplementary Figure S1. (**E**) Relative expression of AID in murine oocytes, granulosa cells, and liver cells as a negative control. AID mRNA expression in granulosa cells was higher than that in oocytes. *Gapdh* glyceraldehyde-3-phosphate dehydrogenase.

First, we immunohistochemically stained murine Peyer’s patches and observed that AID was expressed on B lymphocytes in the Peyer’s patches (Fig. 1A)^19^. In 8-week-old mouse ovaries, AID expression was detected in the oocytes and granulosa cells in the follicles, but not in the surface epithelial cells, interstitial-stromal cells, nor luteal cells in the corpus luteum (Fig. 1A,B). The staining profiles of AID using another anti-AID antibody (Merck Millipore MABF63, clone 328.8) raised against a different antigenic peptide were similar to those detected with the initial antibody (Fig. 1C). The expression of AID was not observed in the ovarian follicles of AID-KO mice, validating the specificity of this assay (Fig. 1C).

In accordance with the results of immunohistological study, mRNA expression of AID in granulosa cells and oocytes was confirmed by RT-PCR (Fig. 1D). RT-qPCR showed that the AID mRNA expression level in granulosa cells was higher than that in oocytes (Fig. 1E). These results suggest that AID is abundantly expressed in the murine ovary, especially granulosa cells.

**Figure 2:**
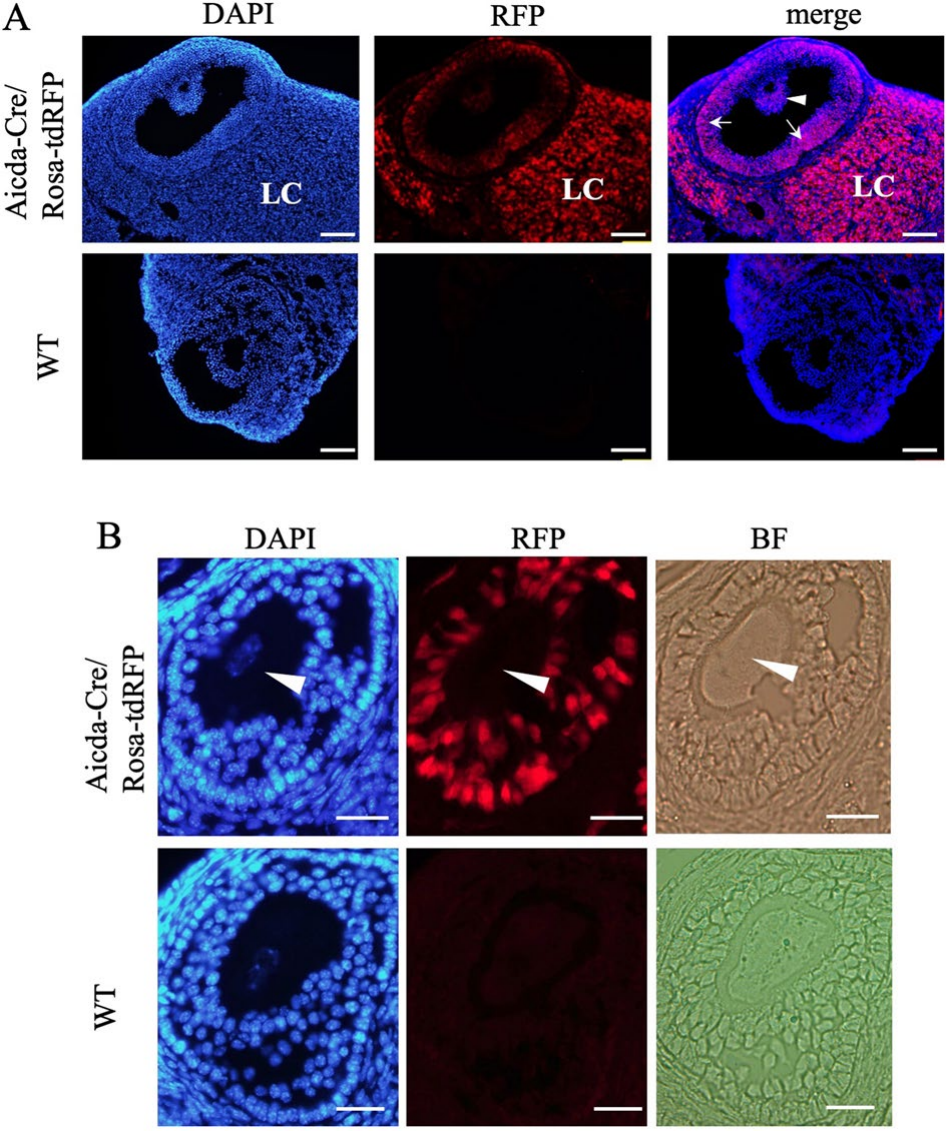
The transcriptional history of AID mRNA in the ovary. (**A**) Immunofluorescence of the 8-week old mouse ovary of an Aicda-cre/Rosa-tdRFP mouse. RFP (red) and DAPI (blue) signals are shown together. RFP was observed in both granulosa cells in the cumulus (arrowhead) and mural (arrows) regions and lutein cells (LC) that are derived from granulosa cells. Bars show 100 μm. (**B**) A magnified view of the follicles. The expression of RFP in oocytes was not detected (arrowhead). Bars show 20 μm.

For further verification, we employed Aicda-Cre/Rosa-tdRFP reporter mice to trace the history of AID expression by tdRFP^7^. However, the expression of RFP was observed in granulosa cells and luteal cells that are derived from granulosa cells (Fig. 2A), but not in oocytes (Fig. 2B).

### Down-regulated genes in the granulosa cells of AID-KO mice

**Figure 3:**
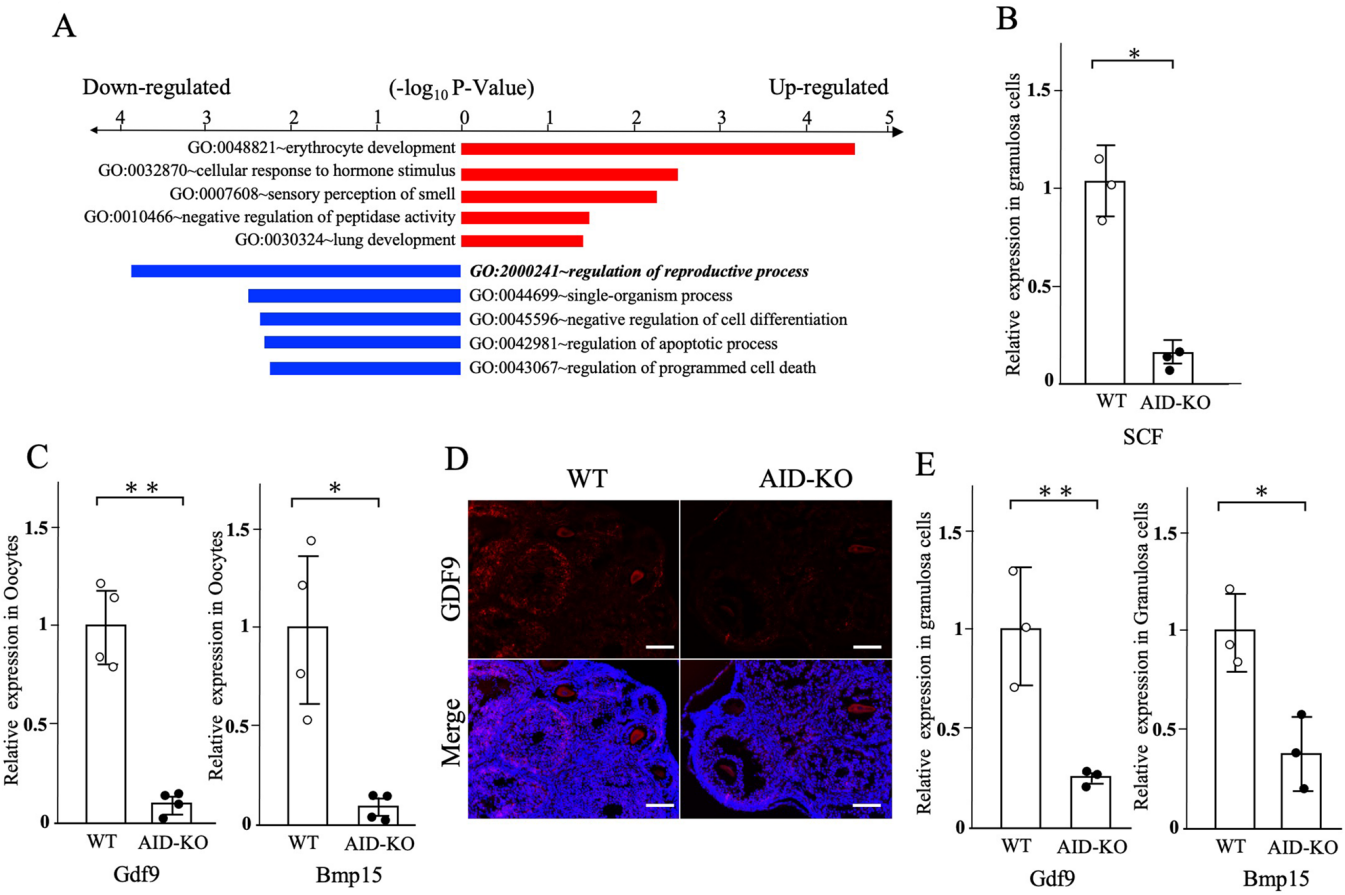
Analysis of GDF-9 and SCF expression in the follicles of AID-KO mice. (**A**) Gene ontology analysis of microarray study. Top 5–7 gene-ontology biological process terms with threshold set at a P-value < 0.05 from up- and down-regulated genes. (**B**) RT-qPCR showed the reduction of mRNA expression of SCF in granulosa cells derived from the AID-KO mouse. Gene expression of Gapdh was used as a control for qPCR analysis. (**C**) RT-qPCR showed that the mRNA expressions of GDF-9 and BMP-15 in oocytes were decreased in oocytes derived from the AID-KO mouse. (**D**) Immunohistological examination showed that the protein expression of GDF-9 was detected in oocytes and granulosa cells in 3-week-old mouse ovaries, whereas its expression was reduced in the AID-KO mouse. Bars show 100 μm. (**E**) RT-qPCR showed that the mRNA expressions of GDF-9 and BMP-15 in granulosa cells were decreased in oocytes derived from the AID-KO mouse. *P < 0.05; **P < 0.01.

To identify the AID-regulated molecules in murine granulosa cells, microarray analysis was performed. Up-regulated genes were defined as those with a fold-change > 2 with highly ranked genes in WAD ranking (304 genes), whereas down-regulated genes were those with a fold-change < 0.5 (274 genes) out of a total of 27,281 genes. Gene ontology biological process term enrichment analyses of the identified up- and down-regulated genes were performed using the Database for Annotation, Visualization and Integrated Discovery (DAVID) with the threshold at a P-value < 0.05, and the top five groups are presented (Fig. 3A).

Since the gene ontology term of regulation of reproductive process was detected in the down-regulated group, we focused on the genes related to follicular formation. Among them, GDF-9 (a secreted member of the transforming growth factor-β superfamily) and SCF (c-Kit ligand) were significantly down-regulated in granulosa cells of AID-KO mice. This reduction was confirmed by RT-qPCR using samples different from those used for the microarray (Fig. 3B).

Since GDF-9 and BMP-15 were reported to be secreted by oocytes, we also examined their mRNA expression in oocytes. RT-qPCR showed that the mRNA expressions of GDF-9 and BMP-15 in oocytes were high and the expressions were decreased in oocytes derived from the AID-KO mouse (Fig. 3C). Immunohistological examination revealed the protein expression of GDF-9 in oocytes and granulosa cells, whereas it was reduced in the AID-KO mouse (Fig. 3D). Since these findings suggest the de novo synthesis of GDF-9 by granulosa cells, we further investigated the mRNA expression of GDF-9 and BMP-15 in granulosa cells. Although the expression rates were low, both mRNA expressions were detected in granulosa cells and their expressions were significantly reduced in the AID-KO mouse (Fig. 3E). These findings suggest that AID regulates folliculogenesis-related genes, in both oocytes and granulosa cells.

### Evaluation of folliculogenesis in AID-KO mice

**Figure 4:**
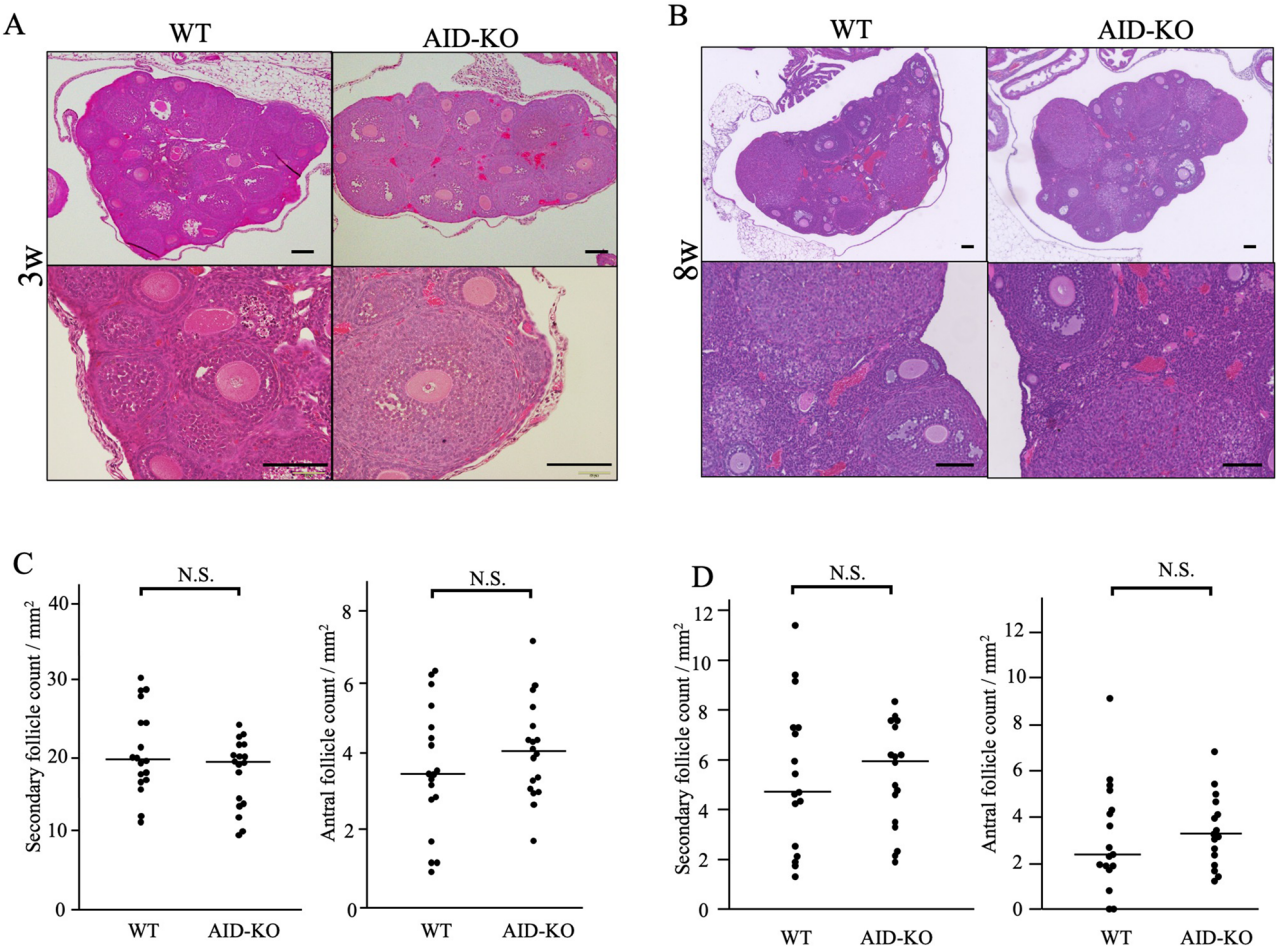
Histological analysis of the follicles in AID-KO mice. (**A**,**B**) Hematoxylin and eosin staining of paraffin-embedded formalin sections of 3-week-old (**A**) and 8-week-old (**B**) wild-type and AID-KO mouse ovaries. In the ovaries of AID-KO mice, there was no abnormality in the structure of the ovaries compared with the wild type. Bars show 100 μm. (**C**,**D**) The number of secondary follicles and antral follicles in 3-week (**C**) and 8-week (**D**) ovaries were calculated as the average number of follicles per ovarian surface area (mm^2^). There was no significant difference in follicular numbers between wild-type and AID-KO ovaries.

The ovarian morphology of AID-KO mice showed no apparent abnormality in the structure compared with the wild type at both 3 and 8 weeks of age (Fig. 4A,B). Since GDF-9 and SCF were reported to be involved in follicular development^20–22^, we counted the number of secondary follicles and early antral follicles, but there was no significant difference (Fig. 4C,D). The average litter size of AID-KO mice (7.0 ± 0.8) was the same as that in the background C57/BL6 mice (data not shown).

### GDF-9 promotes AID and attenuates SCF expression in cultured granulosa cells

**Figure 5:**
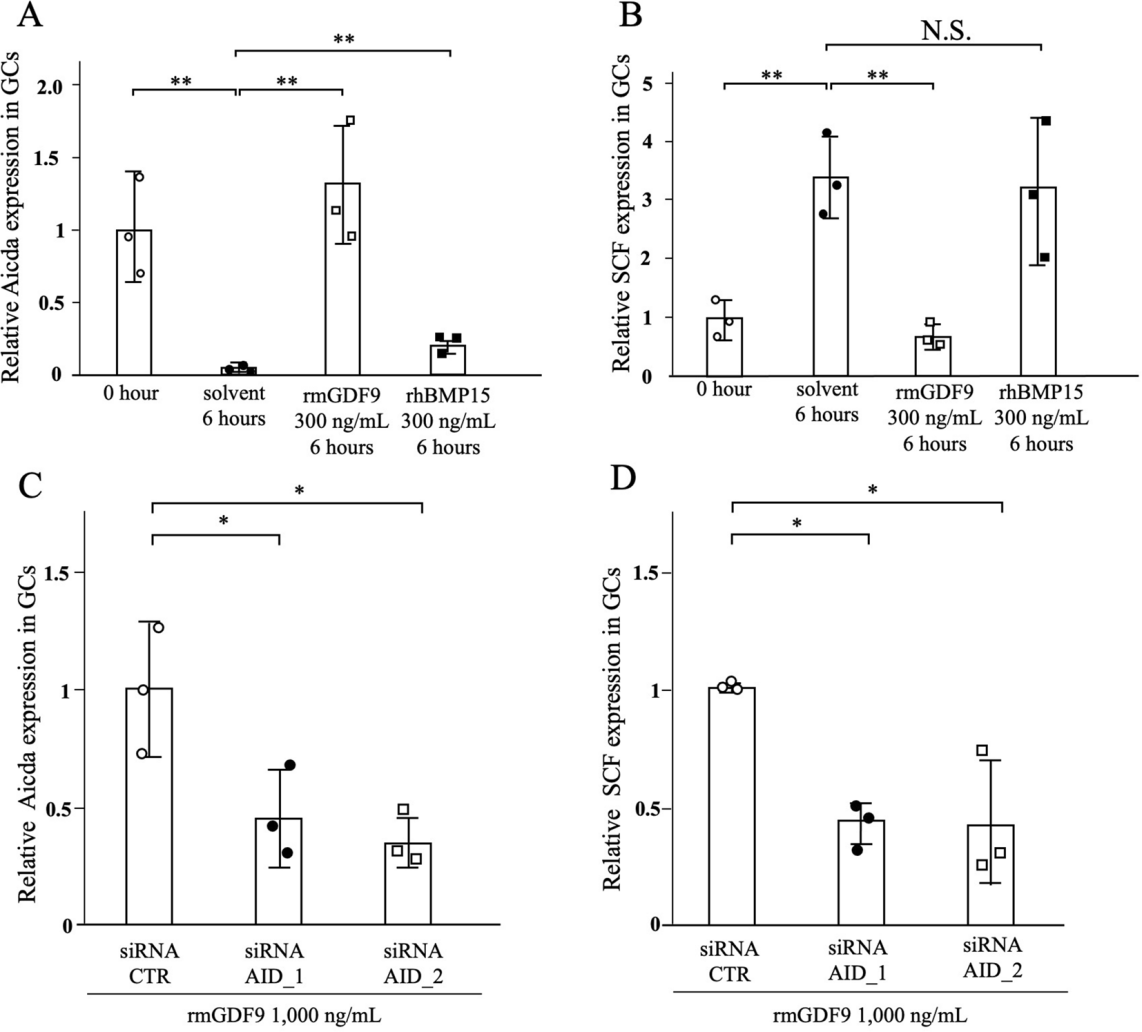
Regulation of SCF expression in cultured granulosa cells by GDF-9 and AID. (**A**–**D**) Granulosa cells isolated from the 3-week ovary of wild-type mice were subjected to the following culture experiments. AID and SCF expression relative to Gapdh in murine granulosa cells assessed by qPCR. (**A**) AID expression was markedly decreased during a 6-h culture. In contrast, AID expression was completely maintained in the presence of GDF-9. BMP-15 also showed enhancing effects on AID expression, but it recovered only a small part of the lost AID expression. (**B**) The mRNA expression of SCF was more than 3-times increased during a 6-h culture. This increase was completely suppressed by GDF-9. BMP-15 showed no effects on SCF expression in cultured granulosa cells. (**C**) In the presence of GDF-9, both siRNA-AID_1 and siRNA-AID_2 significantly suppressed the mRNA expression of AID during a 48-h culture. Under this condition, SCF expression was significantly reduced. *P < 0.05; **P < 0.01.

We next examined how AID expression is regulated in the cross-talk between oocytes and granulosa cells. Since GDF-9 was reportedly a pivotal secretory factor^23,24^, we cultivated primary granulosa cells from 3-week-old wild-type mice. In the absence of GDF-9, there was a rapid and marked decrease in AID expression during a 6-h culture (Fig. 5A). In contrast, in the presence of GDF-9, AID expression was maintained (Fig. 5A). Another oocyte-derived factor, bone morphogenetic protein-15 (BMP-15), also increased AID expression, but recovery of the lost AID expression was slight (Fig. 5A).

On the other hand, the mRNA expression of SCF was more than 3-times increased during a 6-h culture (Fig. 5B). This increase was completely suppressed by GDF-9, as previously reported^25^. In contrast, BMP-15 showed no effects on SCF expression in cultured granulosa cells (Fig. 5B).

### AID attenuates SCF expression in cultured granulosa cells

To further clarify whether intrinsic AID is responsible for SCF expression in granulosa cells, we performed an RNA interference assay. We cultured granulosa cells in the presence of GDF-9, where AID expression was maintained and SCF expression was not increased under the influence of GDF-9. When we performed knockdown of AID mRNA, both siRNA-AID_1 and siRNA-AID_2 significantly suppressed the mRNA expression of AID (Fig. 5C). Under this condition, SCF expression was significantly suppressed (Fig. 5D), which is consistent with the altered gene expression observed in AID-KO mice (Fig. 3). The results suggest that AID expression in granulosa cells was responsible for SCF expression.

### AID did not affect DNA methylation profiles of SCF and GDF-9

**Figure 6:**
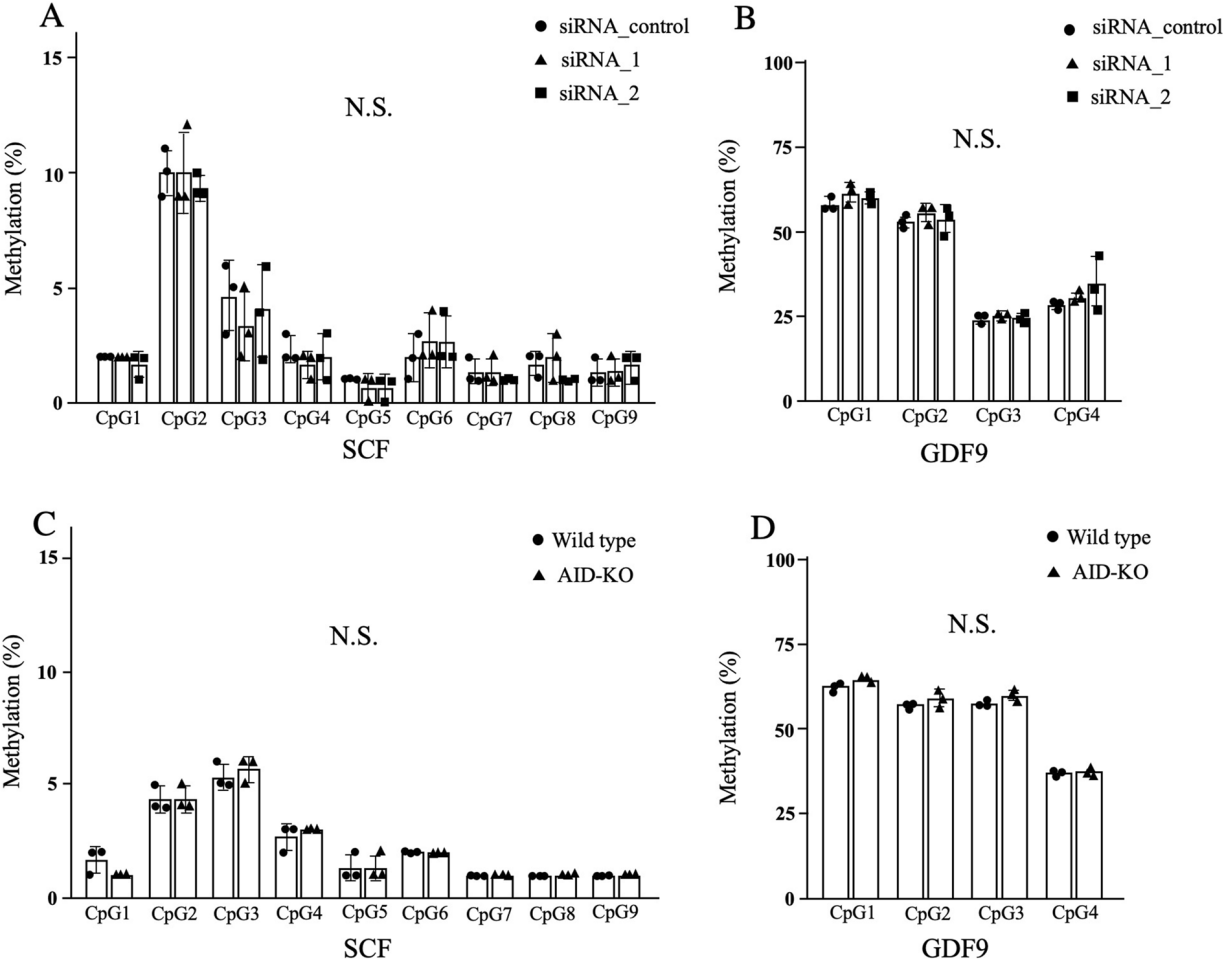
DNA methylation profiles of SCF and GDF-9. (**A**–**D**) The DNA methylation profiles of SCF and GDF-9 using cultured granulosa cells (**A**,**B**) and ovarian tissues (**C**,**D**). In the granulosa cell culture, there were no significant differences in methylation profiles of SCF (**A**) and GDF-9 (**B**) among si-RNA-control, siRNA-AID_1, and siRNA-AID_2 groups. There were also no differences in methylation profiles of SCF (**C**) and GDF-9 (**D**) in the ovarian tissues between wild-type and AID-KO mice.

AID has been suggested to be involved in epigenetic regulation through DNA methylation^9,26^. Thus, we examined the DNA methylation profiles of SCF and GDF-9 using cultured granulosa cells and ovarian tissues. In the granulosa cell culture, gene-knockdown of AID did not affect methylation profiles of SCF and GDF-9 (Fig. 6A,B). We also observed no differences in methylation profiles of SCF and GDF-9 in the ovarian tissues between wild-type and AID-KO mice (Fig. 6C,D).

## Discussion

This study is the first report demonstrating that AID regulates folliculogenesis-related factors, including SCF. GDF-9 and BMP-15, oocyte-secreted soluble factors, were shown to maintain AID expression in granulosa cells, whereas AID is necessary for expression of SCF, which is important for the growth and survival of oocytes. These findings suggest that AID is involved in cross-talk between oocytes and granulosa cells during folliculogenesis.

**Figure 7:**
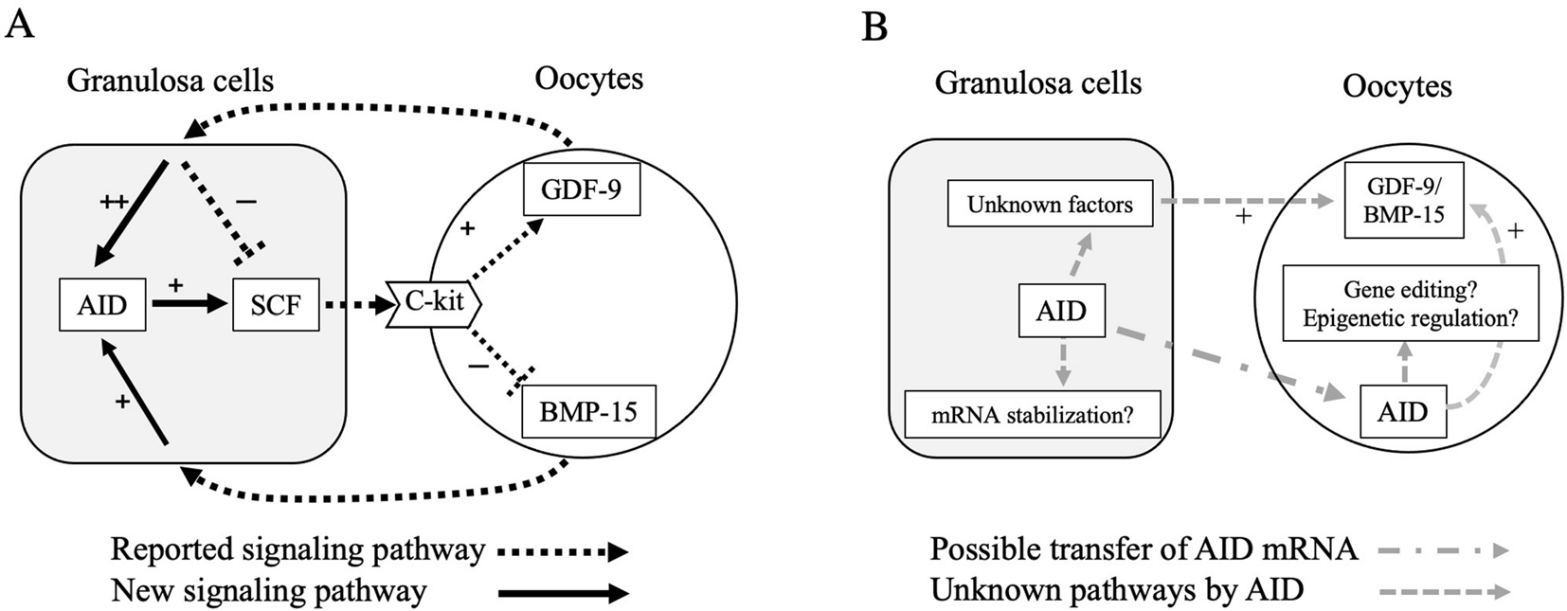
Estimated roles of AID in oocyte-granulosa cell communication. (**A**) Possible involvement of AID in GDF-9/BMP-15 and SCF feedback systems. It was reported that GDF-9 suppresses SCF expression in granulosa cells, whereas SCF promotes GDF-9 expression in oocytes. This study confirmed the suppressive effects of GDF-9 on SCF expression in granulosa cells. It also showed that GDF-9 promotes AID expression and AID enhances SCF expression in granulosa cells, suggesting that AID modulates the GDF-9 and SCF feedback system as a buffering role. On the other hand, SCF was reported to suppress BMP-15 expression in oocytes. In this study, although BMP-15 had no effects on SCF expression, it enhanced AID expression in cultured granulosa cells, suggesting the presence of a negative feedback system between BMP-15 and SCF via AID. (**B**) Agendas of AID in oocyte-granulosa cell communication. AID may contribute to the stabilization of SCF mRNA in granulosa cells. To understand the roles of AID in GDF-9/BMP-15 and SCF feedback systems, AID is speculated to stimulate the expression of unknown factors other than SCF, which induce GDF-9 and BMP-15 expressions in oocytes. On the other hand, AID mRNA in oocytes may originate from granulosa cells. Possible involvement of AID in gene editing and epigenetic reprogramming in oocytes should be clarified in the future.

GDF-9 is a member of the transforming growth factor-beta (TGF-β) superfamily and was reported to be specifically expressed in oocytes^27^. GDF-9 is considered to control the rate-limiting step in the early development of follicles. GDF-9-KO mice showed decrease in proliferation of granulosa cells and arrest of follicle development in the primary stage, leading to failed ovulation and no pregnancies^23,24^. On the other hand, SCF is a ligand for C-kit that is expressed on oocytes and theca cells in murine follicles. SCF was proposed to regulate the growth of oocytes^28,29^ and theca cells^30^. It was reported that murine oocytes developed two-fold in the presence of SCF on in vitro culture, which corresponds to the early stages of oocyte growth^28^. In addition, inhibition of the SCF/c-kit interaction by anti-c-kit antibody induced oocyte death on in vitro culture^29^. SCF was also demonstrated to prevent oocytes in the primordial follicles from undergoing apoptosis in ovarian organ culture^31^. Furthermore, GDF-9 reportedly suppresses SCF expression^32,33^, whereas SCF interacts with oocytes and increases GDF-9 expression as negative feedback^32^. In the GDF-9-KO mouse, SCF expression was increased as high as 32 fold, showing the continuous growth of oocytes^24^. The above reports collectively indicate that GDF-9 and SCF contribute to critical cross-talk between oocytes and granulosa cells during oocyte development and follicular growth (Fig. 7A).

From the expression profiles of GDF-9 and SCF in AID-KO mice, we suggest that AID enhances GDF-9 and SCF expression in follicles. To investigate this possibility, we employed a primary culture of granulosa cells, and confirmed the suppressive effects of GDF-9 on SCF expression, as previously reported^32,33^ (Fig. 5B). Then, we examined the effects of gene suppression of AID by siRNA on SCF expression. This down-regulation of AID reduced SCF expression, supporting the above suggestion that AID enhances SCF expression. This also suggests that AID is involved in critical cross-talk between oocytes and granulosa cells through the GDF-9 and SCF feedback system (Fig. 7A).

Since AID is not a growth factor, the direct mechanism of the decreased expression of SCF in AID-KO mice and AID-knockdown granulosa cells is unknown. To explain this mechanism, we focused on the roles of AID in epigenetic regulation of DNA demethylation. AID reportedly induces demethylation by deaminating 5-methylcytosine to thymine, followed by base excision repair replacing thymine with non-methylated cytosine^9,26^. Nonetheless, AID knockdown did not affect methylation of SCF or GDF-9 (Fig. 6). The mechanism by which AID positively regulates the expression of folliculogenesis-related genes remains to be elucidated. An intriguing possibility is that AID binds to and stabilizes the target mRNAs. APOBEC1 reportedly binds to the 3′ UTRs of mRNA and increases its stability^34,35^. In murine enterocytes, APOBEC1 was reported to bind to cyclooxygenase 2 mRNA, increasing its expression and prostaglandin E2 synthesis^35^. Consequently, AID may contribute to the stabilization of SCF mRNA, but this requires further study (Fig. 7B).

This study showed that AID expression rapidly decreased in the absence of GDF-9 (Fig. 5A), suggesting that continuous stimulation of GDF-9 is necessary to maintain AID expression in granulosa cells. Since GDF-9 is a member of the TGF-β superfamily, the regulation of AID expression in granulosa cells by GDF-9 is supported by our previous findings that AID expression is induced by TGF-β in a murine B-cell lymphoma line^36^. GDF-9-induced AID may counteract the suppressive action of GDF-9 on SCF production. Since granulosa cells in the antral follicles have follicular fluid, the concentrations of GDF-9 and SCF in the follicular cavity are relatively stable. However, in smaller follicles without follicular fluid, changes in GDF-9 production by oocytes rapidly influence SCF production by adjacent granulosa cells. AID may modulate the GDF-9 and SCF feedback system as a buffering role.

Although we detected mRNA and protein of AID in oocytes by RT-PCR and immunohistological staining (Fig. 1), RFP expression was not observed in oocytes of AID-Cre/Rosa-tdRFP reporter mice (Fig. 2). This may be simply attributed to its lower expression level in oocytes, being unable to drive detectable tdRFP expression via Cre recombinase. Alternatively, AID mRNA in oocytes may have an exogenous origin. Since AID is a candidate to control epigenetic reprogramming^6,17,18^, the presence of AID in oocytes is an important issue from the perspective of genetic regulation. Indeed, as follicular EVs were reported to contain granulosa cell-derived mRNA^11^, mRNA of AID may be transported from granulosa cells through follicular EVs^12–15^. In addition, a recent study demonstrated that murine oocytes were directly connected to granulosa cells, by fusing their cellular membrane, which enables the transport of large molecules including GDF-9, from oocytes to granulosa cells^37^. This connection may transport AID protein from granulosa cells to oocytes. The exact origin of AID in oocytes remains to be determined (Fig. 7B).

The expression of BMP-15 in oocytes and granulosa cells was also reduced in AID-KO mice (Fig. 3C,E). In the rat oocyte-granulosa cell co-culture, SCF was reported to suppress BMP-15 expression^32^. This study found no effects of BMP-15 on SCF expression in cultured granulosa cells (Fig. 5B). However, although recovery of the lost AID expression was slight, the BMP-15 enhanced AID expression (Fig. 5A). Considering the promoting effects of AID in SCF mRNA expression in granulosa cells, these findings suggest the presence of a negative feedback system between BMP-15 and SCF via AID (Fig. 7A).

This study had several limitations. First, although AID was shown to regulate folliculogenesis-related genes including GDF-9, BMP-15, and SCF, we could not find evidence that AID knockdown affected fertility under physiological conditions (Fig. 4). This suggests that the reduction of GDF9 and BMP-15 is compensated for by another system. However, we could not detect these candidates from the microarray data. Second, the mechanism of AID-induced changes in gene expression of SCF in granulosa cells and GDF-9 and BMP-15 in oocytes remains unclear. The possible involvement of epigenetic changes in this mechanism should be further investigated (Fig. 7B). Since SCF was reported to have different effects on GDF-9 and BMP-15 expression on oocytes, AID-induced unknown factors other than SCF, which induce GDF-9 and BMP-15 expression in oocytes, should be clarified (Fig. 7B). Third, the phenotype of the ovarian function in the aged AID-KO mouse was not investigated. Since insufficient GDF-9 may promote follicle atresia^38^, additional investigation of the effect of AID deficiency on fertility in the state of aging or under stress conditions should be performed. Finally, the direct effects of AID on oocyte function should be clarified.

In conclusion, this study demonstrated the role of AID in regulating gene expression of follicular cells, suggesting its possible involvement in regulating cross-talk between oocytes and granulosa cells. Further studies are warranted to clarify its role in folliculogenesis.

## Methods

### Mice

C57BL/6 mice at the indicated ages and gestational stages were purchased from Japan Sankyo Lab Service Corporation. AID-KO mice were crossed with C57BL/6 mice for more than 10 generations^2^. The Aicda-cre/Rosa-tdRFP mouse^7^ was prepared by mating Aicda-cre^39^ and Rosa-tdRFP^40^ mice, which were backcrossed at least 10 times on a C57BL/6 background. This mouse showed the transcriptional history of Aicda mRNA as the intracellular expression of RFP (red fluorescent protein). The mice were bred under specific pathogen-free conditions with a natural day/night cycle, fed freely with food and water, and handled in accordance with the guidelines for animal experiments of Kanazawa University. All experimental procedures and housing conditions were approved by the Animal Care and Use Committee of the Kanazawa University Animal Experiment Committee (AP-153522). All studies involving animals are reported in accordance with the ARRIVE guidelines for reporting experiments involving animals^41^.

### Immunostaining methods

The small intestines and ovaries of mice were fixed for 2 h at 4 °C in a fresh solution of 4% paraformaldehyde (Wako, Japan). The samples were then washed in PBS, incubated overnight at 4 °C in a solution of 30% sucrose, and embedded in OCT compound (Sakura Finetek). The tissue segments were sectioned with a cryostat at 8 μm and stained for AID using a tyramide signal amplification system kit with TSA Plus TMR (PerkinElmer)^42^. GDF-9 were assessed by indirect immunofluorescent staining. Immunohistochemical staining was performed using a standard avidin–biotin complex peroxidase method, as described previously^43^. The primary antibodies used in this study were as follows: rat monoclonal anti-AID antibody MAID-2 (eBioscience, 14-5959-80)^44^, rat monoclonal anti-AID antibody (Merck Millipore MABF63, clone 328.8), rabbit polyclonal anti-GDF9 antibody (abcam, ab93862), and rat IgG2b kappa isotype control (eBioscience, 16-4031-81, clone eB149/10H5). The secondary antibodies used in this study were as follows: Peroxidase AffiniPure F(ab’)_2_ donkey anti-rat IgG antibody (H + L) (Jackson Immunoresearch, 712-036-153), Cy3-conjugated goat anti-rabbit IgG antibody (Chemicon, AP132C).

### Follicle count

The number of follicles was counted in three different cross-sections in six ovaries of three mice. A secondary follicle was defined by two or more layers of granulosa cells, and an early antral follicle was defined by two or more layers of granulosa cells with a small antral cavity. Only follicles containing an oocyte were counted. Follicle counts were reported as the average number of follicles per ovarian surface area (mm^2^). The differences in follicle counts were analyzed by the Mann–Whitney U test.

### Isolation of oocytes and granulosa cells

Non-stimulated ovaries from 3-week-old female mice were prepared. We punctured ovaries in the DMEM/F12 culture medium with a 28-gauge needle under a stereomicroscope, and follicles, oocytes, and granulosa cells were released. Oocytes without granulosa cells were isolated manually by a glass micropapillary tube tapered with a burner. If a small number of granulosa cells were attached, they were removed by suction and flushing. Granulosa cells were separated from oocytes and follicles by filtering the suspension through a nylon mesh (40 μm; BD Falcon, Bedford, MA, USA), which allowed granulosa cells but not oocytes or follicles to pass through^45^.

### Culture of granulosa cells

Non-stimulated ovarian follicles from 3-week-old female mice were punctured with a 28-gauge needle, and a mixture of granulosa cells and oocytes was isolated. Granulosa cells were separated from oocytes by filtering the suspension through a 40 μm nylon mesh. The granulosa cells were cultured at 37 °C in a humidified atmosphere (5% CO_2_ and 95% air) in DMEM/F12 supplemented with 0.5% heat-inactivated Fetal Bovine Serum, 5 μg/mL of insulin, 5 μg/mL of transferrin, 5 ng/mL of sodium selenite, and 1% penicillin–streptomycin. Granulosa cells were treated for 6 h with medium containing the solvent or 300 ng/mL of recombinant mouse GDF-9 (739-G9, R&D Systems) or recombinant human BMP-15 (5096-BM, R&D Systems).

### RT-qPCR

RNA extraction and RT-qPCR were performed according to the previously described protocols^43^. Briefly, Total RNA of the mouse ovary was extracted using TRIsure reagent (Bioline, UK). Total RNA from mouse follicles, granulosa cells, and oocytes was extracted using a RNeasy micro kit (Qiagen). Extracted total RNA was treated with amplification-grade DNase I (Invitrogen) and reverse-transcribed using Prime Script reverse transcriptase (Takara Bio Inc., Shiga, Japan) with random primers (nonadeoxyribonucleotide mixture; pd(N)_9_) and oligo dT primers (Takara Bio Inc.). The cDNA was amplified using specific primers (Table 1). Quantitative PCR analysis was performed using SYBR Premix Ex Taq (Takara) on an MX3000 thermo-cycler (Stratagene) following the PCR protocol: a single hot-start cycle at 95 °C for 30 s, followed by 36 cycles of denaturing at 95 °C for 5 s, annealing, and extension at 60 °C for 20 s. Amplification was completed by an additional cycle at 95 °C for 1 min, and PCR products were analyzed by 4% agarose gel electrophoresis and stained with ethidium bromide. Relative gene expression was analyzed using the 2^−ΔΔCT^ method. Results are expressed as the means ± SD of at least two separate experiments using two or more mice. Differences between groups were analyzed for significance by ANOVA or unpaired *t-*tests. *P-*values < 0.05 were accepted as significant.

**Table 1:** Primers for quantitative-PCR.

Name	Sequence (5′–3′)	Product size (bp)
*Aicda*	Forward: CTGTGAAGACCGCAAGGCTGAG	161
	Reverse: AATTTTCATGTAGCCCTTCCCAGG	
*Gdf9*	Forward: CCTCTACAATACCGTCCGGC	115
	Reverse: CACCCGGTCCAGGTTAAACA	
*Kitl* (SCF)	Forward: CATCCATCCCGGCGACATAG	183
	Reverse: CATCCATCCCGGCGACATAG	
*Gapdh*	Forward: TGAAGCAGGCATCTGAGGG	102
	Reverse: CGAAGGTGGAAGAGTGGGAG	

*Gapdh* glyceraldehyde-3-phosphate dehydrogenase, *Kitl* kit ligand.

### Microarray analysis

Total RNA from mouse granulosa cells was extracted for microarray analysis. Using 3-week-old female wild-type and AID-KO mice, follicles were punctured with a 28-gauge needle and granulosa cells were separated from oocytes by filtering the suspension through a 40-μm nylon mesh. Microarray experiments were performed using SurePrint G3 Mouse 8 × 60 K ver. 2.0. Data analysis was performed using GeneSpring GX software (Agilent Technologies). In brief, raw intensity data were calculated with Agilent Feature Extraction 12.0.3.1 and normalized as a 75 percentile shift according to the protocol.

Differential Expressed Genes were extracted from normalized microarray intensity data using the weighted average difference (WAD) ranking method^46^. This method is a statistical approach based on the fold-change method that uses not only the difference in gene expression, but also the signal intensity in microarrays. Fold-change was calculated by dividing the AID-KO value by the wild-type value.

### RNA interference assay

AID-siRNA (MSS235859-60, Thermo Fisher Scientific) and negative control siRNA (AM4635, Ambion) were used. siRNA transfections were carried out by the reverse transfection method with Lipofectamine RNAi MAX according to the manufacturer’s instructions. In brief, 20 pmol siRNA and 1 μL of Lipofectamine RNAi MAX were incubated in 100 μL Opti-MEM medium (Thermo Fisher Scientific) for 20 min at room temperature in 24-well plates, and then 500μL of cell suspension (2.0 × 10^5^ cells) was added to the siRNA–RNAi MAX complex. The cells were incubated for 48 h at 37 °C in a humidified atmosphere containing 5% CO2 and 95% air in the DMEM/F12 described above with 1000 ng/mL of recombinant mouse GDF-9.

### DNA methylation assay

DNA methylation assay was performed according to the previously described protocols^47^. Briefly, DNA from individual mouse ovaries and granulosa cells was extracted and bisulfite converted using the EZ DNA Methylation Gold Kit (Zymo Research, Orange, CA USA) according to the manufacturer’s instructions and was eluted in 20 μL of water. Pyrosequencing primers were designed for each target of interest using PyroMark Assay Design 2.0 software (Qiagen Inc., CA, USA) to amplify the bisulfite-modified target region. PCR reactions were carried out with 25 ng of bisulfite-converted DNA using the PyroMark PCR kit (Qiagen Inc., CA) in a final volume of 25 µL containing 12.5 µL 1 × PyroMark PCR Master Mix, 2.5 µL 1 × CoralLoad Concentrate, 0.5 µL of each primer in a final concentration of 0.05 µM, and 8 µL of water. Amplification conditions were as follows: 95 °C for 15 min, 45 cycles of 94 °C for 30 s, 56 °C for 30 s, and 72 °C for 30 s, and finally, 72 °C for 10 min. The pyrosequencing was performed using a Pyromark Q24 pyrosequencer (Qiagen Inc., CA) following the manufacturer's recommended protocols. Pyromark Q24 software was used to calculate the percent methylation for each CpG site. The results are displayed as a pyrogram with the methylation percentage.

### Statistical analysis

In RT-qPCR studies, results are expressed as the means ± SD of at least two independent experiments using two or more mice. The significant differences between groups were analyzed by ANOVA followed by Dunnett or unpaired *t-*tests using Microsoft Excel software. The numbers of follicles were counted in three different cross-sections in six ovaries of three mice. The differences were analyzed by the Mann–Whitney U test (SPSS Statistics version 25.0, IBM, USA). *P-*values < 0.05 were regarded as significant.

## Supplementary Information

Supplementary Information
