# Supplementary figures and images for: Activation-induced cytidine deaminase is a possible regulator of cross-talk between oocytes and granulosa cells through GDF-9 and SCF feedback system
Source: Sci Rep. 2021 Feb 15;11:3833. doi: 10.1038/s41598-021-83529-x (PMC7884688; doi:10.1038/s41598-021-83529-x)

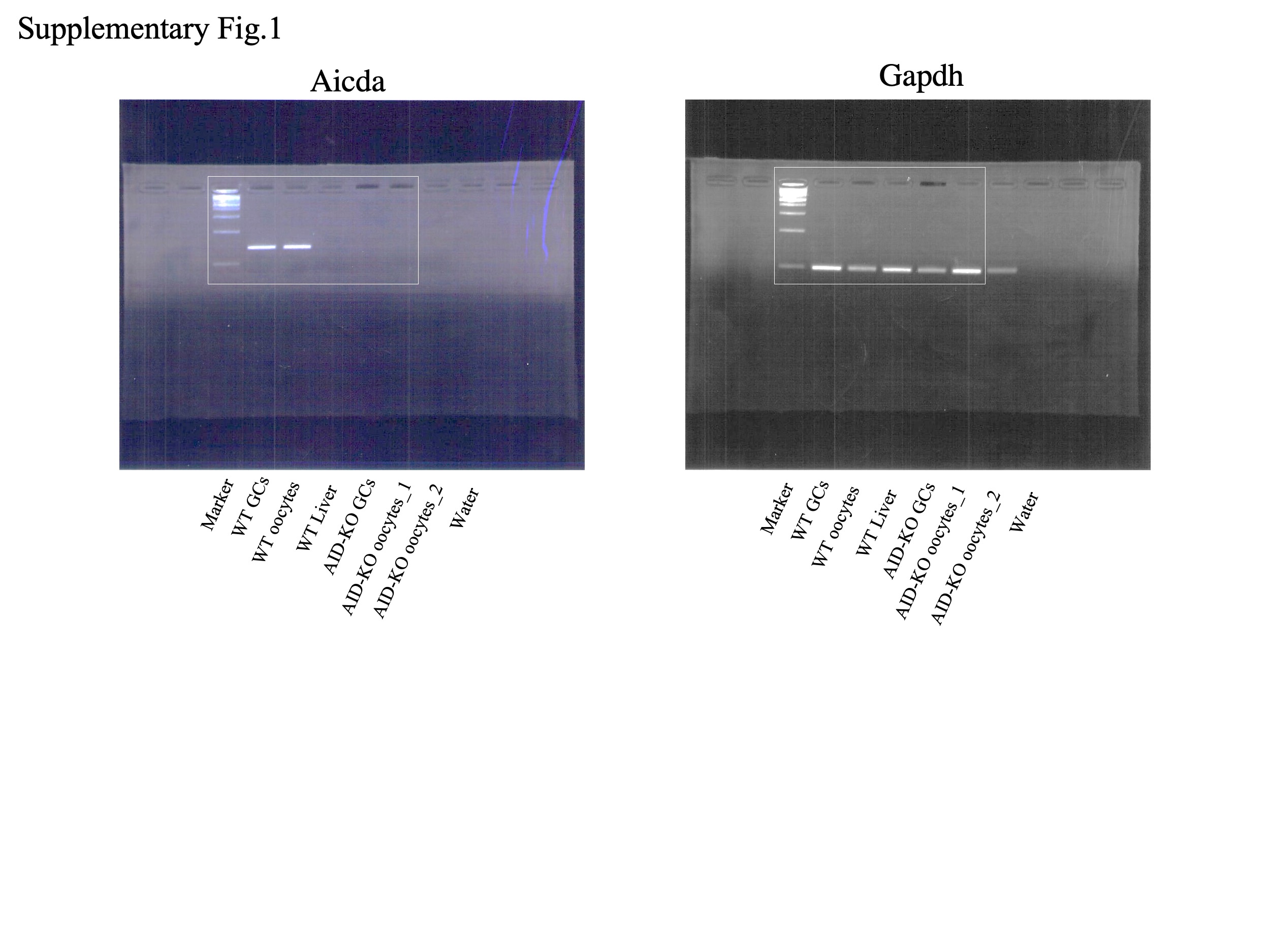

Supplement: Supplementary file 1 — Supplementary Information [file 41598_2021_83529_MOESM1_ESM.jpg]
